# Novel Insight Into the Molecular and Metabolic Mechanisms Orchestrating IL-17 Production in γδ T Cells

**DOI:** 10.3389/fimmu.2019.02828

**Published:** 2019-12-03

**Authors:** Xu Chen, Samantha Morrissey, Fuxiang Chen, Jun Yan

**Affiliations:** ^1^Department of Clinical Immunology, Shanghai Ninth People's Hospital, Shanghai Jiao Tong University School of Medicine, Shanghai, China; ^2^Immuno-Oncology Program, Division of Immunotherapy, Department of Surgery, James Graham Brown Cancer Center, University of Louisville, Louisville, KY, United States; ^3^Department of Microbiology and Immunology, School of Medicine, University of Louisville, Louisville, KY, United States; ^4^Faculty of Medical Labratory Science, Shanghai Jiaotong University School of Medicine, Shanghai, China

**Keywords:** innate immune cells, γδ T17 cells, transcriptional regulation, metabolic reprogramming, cancer immunotherapy

## Abstract

Increasing evidence has demonstrated that IL-17-producing γδ T cells (γδ T17) play a tumor-promoting role in a series of cancers via various mechanisms in mice and human cancers, though the relationship between γδ T17 and human tumors has yet to be extensively characterized and established. Molecular signals such as intrinsic cascade, environmental cues and cellular metabolic pathways including nutrient uptake and utilization in γδ T17 cells are significantly important for their activation, differentiation, and function. Understanding the molecular mechanisms and metabolic pathways of γδ T17 cells in both the physiological setting and tumor environment would contribute to the development of therapeutic approaches or drugs targeting γδ T17 for immunotherapy in cancers.

Innate γδ T cells are a complex cohort of cells with diverse functionality in both physiological and disease conditions. While γδ T cells can be subdivided into multiple different subsets based on expression profile, they can largely be classified into two main functional groups- IFN-γ producing (γδ T1) and IL-17-producing (γδ T17) γδ T cells (1). While both direct and indirect antitumor effects of γδ T cells have been reported, the emerging consensus within the field suggests that the γδT17 subset possess pro-tumorigenic characteristics mainly mediated by IL-17A production. Specifically, IL-17A from γδ T cells has been shown to induce angiogenesis within the tumor microenvironment (TME), and increase recruitment of immunosuppressive cell types like myeloid-derived suppressor cells (MDSCs), neutrophils, and tumor associated macrophages (2). Therefore, given the prominent role of IL-17 in tumorigenesis, it is important to better understand the mechanisms responsible for regulating IL-17 secretion in cancer and at baseline in normal physiological conditions. Here, we review the mechanistic drivers and metabolic pathways controlling IL-17 production in γδ T cells in hopes to provide new approaches to cancer treatment by targeting γδ T17.

## γδ T17 in Physiological Conditions and Tumor Environment

γδ T cells are a unique subset of cells that combine conventional T cell adaptive immune features with rapid innate-like responses. Given this specific functionality, γδ T cells, particularly γδ T17, are often found in barrier and mucosal sites like the skin, oral mucosa, gut lamina propria, and lung in both mice and humans (3–7). Murine γδ T17 have been shown to play important roles in tissue homeostasis, anti-infectious pathogen clearance and body temperature maintenance despite an overall low abundance in the body (3, 4, 8). Furthermore, in mice, γδ T17 cells contain Vγ4^+^, Vγ6^+^ and minor Vγ1^+^ subsets, and characteristically express RORγt, c-Maf, and CCR6 (5–7, 9, 10). While both Vγ4^+^ and Vγ6^+^ subsets have been shown to produce IL-17, Vγ6^+^ cells produce IL-17 exclusively, and augment production in the context of cancer or inflammation whereas Vγ4^+^ are more heterogeneous and can produce IFN-γ or IL-17 depending upon environmental context. The difference in cytokine profile is likely a result of differential development and peripheral regulation. It has been shown that development of Vγ6^+^ is restricted to a functional embryonic wave that is dependent on the fetal thymus whereas Vγ4^+^ are more complex and are heavily reliant on bone marrow progenitors (5). Further studies revealed differential homing patterns in the dermis and lung, and to a less extent in the lymph nodes (LN) and spleen, between the two subsets with Vγ6^+^ often outcompeting Vγ4^+^. However, in spontaneous and transplantable cancer models, both subsets have been identified as prominent IL-17 producers (2, 11, 12). While the protumoral characteristics of γδT17 cells are not the topic of this review it should be noted that other prominent tumor promoting roles include functioning like regulatory T(Treg)/T helper2 (Th2) like cells, interfering with dendritic cell effector functioning, and inhibiting T cell effector functioning via the programmed death-1 (PD-1)-programmed death ligand-1 (PD-L1) pathway (2, 13, 14).

**Figure 1:**
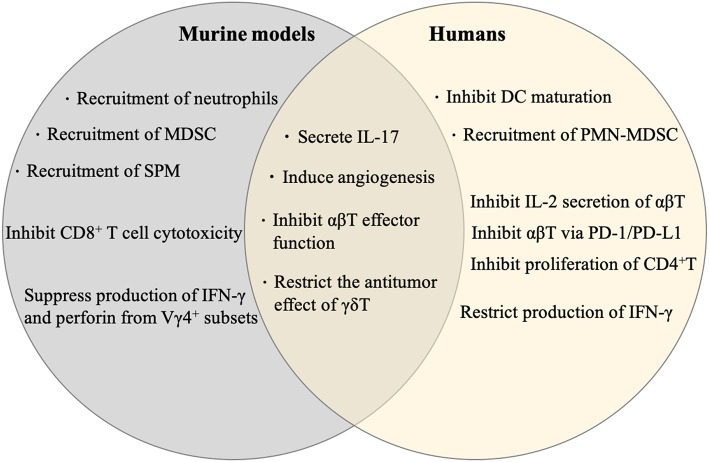
Proposed mechanisms (differential and shared pro-tumor mechanisms) by γδ T17 in murine models and humans (2, 11–13, 16, 18, 19).

In humans, γδ T cells mainly consist of tissue-resident Vδ1^+^ and peripheral Vδ2^+^ subsets. Tissue-resident Vδ1^+^ γδ T cells are often found in epithelial layers and play significant roles against infections and tissue integrity (15). The Vδ2^+^ (mostly Vγ9^+^) population is more heterogeneous and can respond to a variety of pathogens (16). Both of these subsets can produce IFN-γ upon activation, but the Vδ1^+^ is reported to produce more IL-17 in some cancers like pancreatic ductal adenocarcinoma and colorectal carcinoma despite limited overall characterization studies (16, 17). Like their murine counterparts, human γδ T17 cells have been shown to promote tumor growth in a variety of human cancers including colorectal cancer, lung cancer, breast cancer and pancreatic ductal adenocarcinoma. (13, 16, 18, 19). However, a recent study revealed that breast-resident Vδ1^+^ cells differentially produce IFN-γ, not IL-17, and that breast-resident Vδ1^+^ are associated with remission in triple-negative breast cancer (20), thus demonstrating the complexity of human γδ T17. There are some conserved protumor effectors mechanisms between murine and human γδ T17 cells including increased angiogenesis and inhibition of αβT cells, but other distinct mechanisms have been identified in humans as shown in Figure 1 (2). While further characterization of the role Vδ1^+^ play within the human tumor microenvironment of different cancers is warranted, a limited collection of evidence suggests these cells convey an immunosuppressive pro-tumoral phenotype.

Overall, Vγ6^+^ and to an extent Vγ4^+^ cells in mice and Vδ1^+^ cells in some human cancers like colorectal carcinoma and pancreatic ductal adenocarcinoma are considered to be tumor progressing γδ T17. However, further studies are needed for exploring the human γδ T17 and its relationship with other cancer types. The molecular mechanisms governing IL-17 production are often specific to each particular cell type and will be covered individually in the next section. Therefore, understanding the molecular mechanisms and metabolic pathways orchestrating γδ T17 cells would help contribute to developing new immunotherapies in cancers.

## Molecular Signals Orchestrating IL-17 Production in γδ T Cells

**Figure 2:**
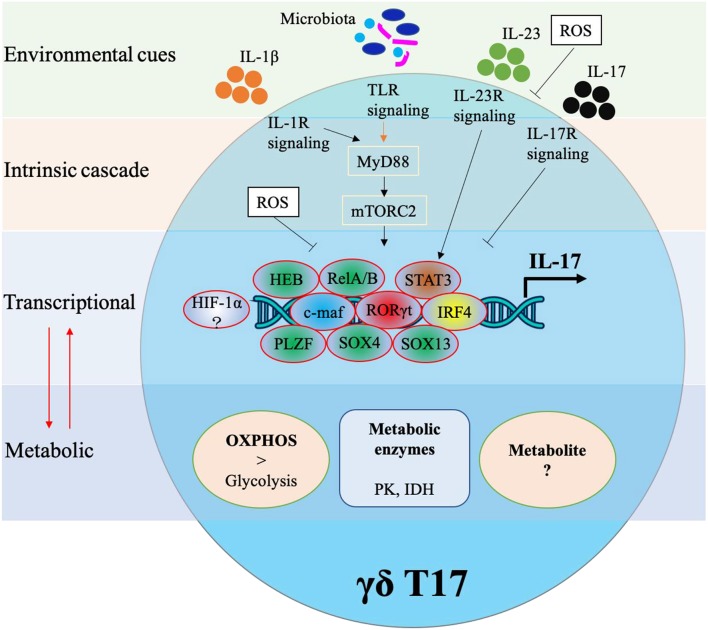
Transcriptional and metabolic regulation of IL-17 production in murine γδ T cells.

Cytokine production in γδ T cells can be preprogrammed in the embryonic thymus or induced in the periphery. Either way, cytokine production in γδ T cells requires complex networking. As shown in Figure 2, IL-17 production in murine γδ T cells is controlled by various transcriptional factors and also regulated by external stimulations. In this next section, we will identify specific transcription factors associated with IL-17 production in γδ T17.

### Transcription Factors (TFs)

One of the most prominent transcription factors associated with IL-17 production is RORγt. It is known to control IL-17 production in Th17 cells and similarly has been found to be a core transcription factor for IL-17 production in murine CD27^−^ γδ T cells (1, 21). However, unlike Th17 cells, RORγt expression alone in γδ cells is enough to induce IL-17 whereas Th17 cells often need collaborative signals from TGF-β, IL-6, or IL-21. Interestingly, RORγt can maintain its own expression by binding at the *Rorc* CNS+10 (a conserved non-coding sequence located 10 kb from the Rorc(t) transcription start site) (10). RORγt usually cooperates with other TFs such as STAT3, IRF4, and BATF in Th17 differentiation (22), but what specific factors regulate RORγt in the context of γδ T17 cells remains largely unexplored.

One TF that has been identified to augment RORγt driven IL-17 production in γδ T cells is c-Maf. The AP-1 TF c-Maf has been found to predominantly bind at *Rorc* CNS+10, thus stabilizing RORγt expression (10). c-Maf also inhibits the binding of TCF1 (a negative regulator of RORγt) to *Rorc* to further support the expression of RORγt. Furthermore, c-Maf regulates chromatin accessibility thus increasing the probability of RORγt binding. Interestingly, c-Maf can also promote γδ T17 through a RORγt-independent mechanism via directly regulating *Blk* and *Tcf7* (encoding TCF1).

It has been reported that IRF4, RORα and BATF are not required in IL-17 production of γδ T cells (22, 23), but a recent study revealed that IRF4 played a significant role in IL-17 production of murine dermal γδ T cells including Vγ4^+^ and Vγ6^+^ subsets (24). Specifically IRF4 links IL-1R and IL-23R signaling pathways to IL-17 production. One of the main transcription factors upstream from IRF4 is STAT3 (24). STAT3 activation is crucial for RORγt expression in Th17 cells and also significant for IL-17 production in γδ T, though some subsets of γδ T have been found to be independent of STAT3 (24–26). Specifically, IL-23-induced STAT3 signaling plays a pivotal role in the production of IL-17 in dermal Vγ4^+^ but not in Vγ6^+^ subsets (24). Similar to STAT3, some other TFs have been found to control IL-17 production in γδ T subsets. The high-mobility group (HMG) TFs SOX4 and SOX13 were required for Vγ4^+^ subsets, and SOX4 was an essential regulator of *Rorc* whereas SOX13 regulated *Blk* expression (27). Moreover, their upstream TF HEB (HeLa E-box binding protein) regulated the expression of SOX4 and SOX13 by interacting with the regulatory region of DNA (~25 kb 5′ of the *Sox4* transcriptional start site and predicted 32 kb 5′ of the second start site of the *Sox13* locus) (28). The promyelocytic leukemia zinc finger (PLZF) TF was required for IL-17 secretion and maturation in Vγ6^+^ subsets, but the detailed molecular mechanism remains to be explored (29). Interestingly, a recent report found that PLZF^+^ γδ T cells promote a thermogenic response via directly producing cytokines such as IL-17 and TNF-α and indirectly maintaining catecholamine sensitivity (8). Collectively, apart from the core transcription factor RORγt, universal transcription factors (c-Maf, IRF4), which play a role in all γδ T17 subsets, and partial transcription factors (STAT3, HEB, SOX4, SOX13, and PLZF), which play a role in some γδ T17 subsets, work in concert or independently for the controlling of IL-17 production in different γδ T subsets.

### Cell Surface Receptors and Cellular Intrinsic Cascade

Mouse γδ T17 cells express a variety of innate receptors including TLR1, TLR2, and dectin-1, but not TLR4. Activation of TLRs and dectin-1 leads to increased IL-17 production in γδ T cells (30), solidifying their role as non-histocompatibility complex restricted lymphocytes. Moreover, γδ T17 cells express IL-23R and IL-1R which, following IL-1β with IL-23 stimulation, enhances IL-17 gene expression and protein production (6, 31, 32), suggesting that both PAMP and cytokine receptors play significant roles in the IL-17 production in γδ T cells. In fact, the indispensable roles of IL-1R and/or IL-23R in γδ T17-mediated diseases such as experimental autoimmune encephalomyelitis (EAE) and psoriasis-like skin inflammation have been validated in murine models (6, 31, 32). By exploring the molecular mechanism underlying the IL-1β-IL-17 axis, IL-1R-MyD88-mTORC2 was found in both dermal Vγ4^+^ and Vγ6^+^ subsets, which primarily produced IL-17 (24). MyD88 is an adaptor protein which is required for most TLR signaling and therefore is necessary for TLR signaling-induced expansion and cytokine production of γδ T17. However, the detailed cascade or mechanism of TLR signaling in IL-17 production of γδ T remains to be understood. The cytokine IL-23 differentially enhanced IL-17 production via the IL-23R/STAT3/IRF4 pathway in dermal Vγ4^+^ and via the IL-23R/RelA/IRF4 pathway in dermal Vγ6^+^ (24). These results suggested that IL-1β and IL-23 synergistically induced IL-17 production albeit through distinct pathways. Unexpectedly and of note, IL-17 itself is a negative regulator of γδ T17 as *Il-17r* knockout increased the IL-17 production of γδ T from cervical LN and inguinal LN (5, 33). The mechanism behind this negative feedback loop have yet to be determined.

Furthermore, both the classical and non-canonical NF-κB signaling pathways are important for γδ T17. RelA or RelB conditional deficiency leads to reduction of γδ T17 cells through reducing *Il-17* and *Rorc* expression at the transcriptional level, and p52, not p50 was also required for IL-17 production (34). NF-κB-inducing kinase (NIK), which is required for non-canonical NF-κB signaling, was essential for IL-17 production as NIK depletion led to impaired *Rorc* and *Sox13* expression (35). Notch signaling and its downstream target Hes1, one of the basic helix-loop-helix (bHLH) proteins, were essential for the IL-17-producing function of mature γδ T cells in the periphery (36). It should be noted that the majority of mouse γδ T17 cells get functional preprogramming in the thymus, and factors like Notch signaling that have influence on the development of γδ T17 may affect the IL-17 production of γδ T cells.

It has been reported that TCR signaling is a major determinant of the functional differentiation of γδT cells in the thymus. Strong TCR signaling determines the lineage fate of the earliest progenitor T cells toward the γδ subset (37, 38). However, little is known about how γδ TCR engagement drives IL-17 production in mature γδ T cells. A recent study identified the Syk/PI3K/Akt pathway as one means to control the development of γδ T17 (39). Additionally, c-Maf regulation of RORγt which was noted above is dependent on γδ TCR ligation with too strong a signal limiting c-Maf expression (10). Overall, a variety of signaling pathways including PAMPS, cytokines, Notch and γδ TCR ligation have been shown to orchestrate IL-17 production in murine γδ T cells.

It is important to keep in mind that human γδ T17 are different from murine γδ T17. Little is known about human γδ T17 in terms of origin, differentiation, and transcriptional regulation. While they express RORγt, CCR6, IL-23R, and IL-1R like their murine counterparts (40), human γδT17 cells may originate from naive precursors in the peripheral blood when activated with pathogen products and IL-23 stimulation (41). STAT3 deficiency in humans leads to a loss of IL-17 in γδ T cells though it does not affect the frequency of γδ T cells (42). RORγt inhibition could selectively target IL-17-producing innate cells including γδ T17 in patients with spondyloarthritis, demonstrating that RORγt antagonism could be a promising therapeutic approach (43). It has been reported that a cocktail of cytokines could maintain and differentiate human γδ T17 (44, 45), however a detailed mechanism has not been elucidated. Therefore, more information is needed for a better understanding of the mechanism underlying the development and function of IL-17-producing γδ T cells in humans.

## Cellular Metabolism and Metabolic Reprogramming of γδ T17 Cells

Cellular metabolism is coming to the forefront as an important indicator of cellular function. Specifically, cells often undergo metabolic reprogramming in the context of disease which ultimately affects the cells natural effector function. Naive T cells, for example, are quiescent with low metabolic demands and mainly rely on oxidative phosphorylation (OXPHOS) for ATP, while activated T cells dramatically upregulate glycolysis and downregulate mitochondria-dependent fatty acid oxidation (FAO) and pyruvate oxidation through the TCA cycle (46). This hypermetabolic reprogramming meets the demands for T cell activation in the context of infection or autoimmunity. Conversely in cancer, exhausted T cells are often found to be in a hypometabolic state which corresponds to their overall suppressed phenotype. The relationship between metabolism and T cell function has been summarized in detail elsewhere (47–49). However, how metabolism impacts the effector function and differentiation of γδ T cells has been largely unexplored. Here, we will review what is known about the factors orchestrating γδ T17 cell metabolism and how they may contribute to the understanding of γδ T cell metabolism for immunotherapy.

### Metabolic Pathway and Cellular Function in γδ T Cells

Just like their cytokine profiles, the metabolic signatures of γδ T cells differ between the two main subsets. γδ T1 cells have a preference for glycolytic metabolism while γδ T17 rely more on OXPHOS (24). Classically, this fits with the pattern that pro-inflammatory cells rely more heavily on glycolysis while anti-inflammatory/pro-tumoral cells mainly utilize OXPHOS or fatty acid metabolism. Inhibition of glycolysis with 2-deoxy-D-glucose (2-DG) showed no impact on the IL-17 production of dermal γδ T, while inhibition of isocitrate dehydrogenase (IDH) or pyruvate kinase (PK) significantly reduced IL-17 production of dermal γδ T cells in a dose-dependent manner (24), suggesting that the citric acid cycle (TCA) and OXPHOS play a significant role in IL-17 production. One of the key regulatory enzymes that serves as a central mediator between extrinsic signals like cytokines and environmental cues to cell-intrinsic metabolism is mammalian/mechanistic target of rapamycin (mTOR) (24, 50–52). There are two distinct mTOR complexes, mTORC1 and mTORC2 which contain scaffold proteins Raptor or Rictor, respectively. In dermal γδ T cells, stimulation with IL-1β and/or IL-23 directly activates mTOR resulting in increased IL-17 production. However, conditional knockout (cKO) of Rictor leads to reduced IL-17 production in both Vγ4^+^ and Vγ6^+^ subsets while Raptor cKO has no influence on IL-17 production of dermal γδ T (24). Neither Raptor cKO nor Rictor cKO impacted 2NBDG uptake in T-cell indicating that mTOR signaling specifically impacts OXPHOS. Overall, these results suggest differential roles of mTORC1 and mTORC2 in the regulation of γδ T17. Rictor cKO alone leads to more dysfunctional mitochondria and reactive oxygen species (ROS) production, which implies that functionally respiring mitochondria are essential to γδ IL-17 production. Additionally, ROS negatively feeds back to IL-17 production in γδ T cells since treatment with ROS inhibitor N-acetyl-L-cysteine (NAC) rescued the IL-17 production (24). A recent report found that murine Vγ6^+^ γδ T17 cells displayed low expression of ROS neutralizing antioxidant glutathione. Consequently, tumor-associated neutrophils were then able to inhibit the proliferation of murine Vγ6^+^ γδ T17 cells via induction of ROS, suggesting novel approach for targeting the neutrophil/ROS/γδ T17 axis in the tumor microenvironment (53).

While OXPHOS is most often mentioned in the context of metabolism and ATP generation, a secondary less recognized function is thermogenesis, or the generation of body heat. Uncoupling of OXPHOS from ATP synthesis via uncoupling protein 1 (UCP1) results in the potential energy of the electron transport chain being converted into thermal energy or heat. A recent study found that γδ T17 cells play an important role in regulating this process (8). γδ T cells and IL-17 have been linked to maintaining homeostasis through T regulatory cell (T_reg_) mediated thermogenesis. T_regs_ dominate the CD4^+^ compartment within adipose tissue and generate large amounts of IL-33, a cytokine critical for body temperature regulation. They found that Vγ6^+^ accumulate in adipose tissue over time and can directly influence non-shivering thermogenesis. Specifically, the CD27^−^ γδ T cells through IL-17 mediate expansion of adipocyte resident T_regs_ which secrete the IL-33 necessary to induce UCP1 thermogenic effector function. Mice deficient in Vγ6^+^ or global IL-17KOs were unable to successfully regain thermostatic equilibrium following cold challenge indicating the importance of γδ generated IL-17 in thermogenesis.

The generation of IL-17 in γδ T cells is closely tied with mitochondria function and oxidative phosphorylation capacity. It has been shown that γδT17 cells preferentially utilize OXPHOS over glycolysis and reduction in OXPHOS capacity correlates with decreased mitochondria function and concomitantly decreased IL-17 production. Furthermore, decreased IL-17 and Vγ6^+^ cells in adipose tissue results in decreased uncoupling of OXPHOS and overall inability to thermoregulate in response to environmental thermo-fluctuations. However, further investigation into how OXPHOS and TCA metabolism regulates IL-17 production is warranted. It remains unclear how pre-programming in the thymus affects metabolism, whether there are any differences in metabolic condition between activated γδ T17 and resting γδ T17, and how γδT17 metabolism differs from Th17 cells. Previous studies have shown that TF Myc is critical for glycolysis and glutaminolysis in activated T-cells, and HIF-1α is important for Th17 differentiation (50, 54). How these transcription factors impact γδT17 development, particularly in the context of a hypoxic tumor microenvironment, has yet to be explored. In summary, limited information about the metabolism of γδ T17 has been reported. With the advance of new technologies such as systems metabolomics and single cell sequencing, more details on the metabolism of γδ T17 could be revealed.

## Crosstalk Between γδ T17 and Other Cells and Microorganism in TME

We are just beginning to acquire a better understanding of the molecular mechanisms and metabolic regulations governing γδ T17. However, the question becomes even more complex when taking into account the interaction between γδ T17, other cells, and microorganisms in the TME. A recent report characterizing the interaction between microbiota, immune cells and γδ T17 in lung adenocarcinoma microenvironment found that altered commensal microbiota promote lung cancer by activating γδ T17 through PMAP/MyD88-mediated production of IL-1β and IL-23 from myeloid cells. (18). These tumor-associated γδ T17 showed a distinct transcriptional profile from that of spleen indicating environmental context is important for dictating function. This report builds on the previous suggestion that intact commensal microbiota and γδT17^+^ in the lungs are necessary to mount sufficient immune responses against B16/F10 or LLC tumor challenge (55). Therefore, further investigation into the drivers behind altered lung commensal microbiota is warranted along with identification of pathologic species to help tease apart the role of γδ T17 in health and disease. Whether the tumor-associated γδ T17 display a different metabolic condition as compared to other γδ IL-17 producing subsets remains unknown. In murine models, oral microbiota drives γδT17 proliferation and activation via CD103^+^ DCs' cell-to-cell contact (33), while in the human colorectal cancer microenvironment, γδT17 were polarized by microbiota-activated inflammatory DC-producing IL-23 and played a pivotal role in the cancer progression (16). A key tumor promoting phenotype of IL-17 is to recruit tumor-associated neutrophils or PMN-MDSCs to the local microenvironment. However, tumor-associated neutrophils can inhibit γδT17 via ROS production (53). With more emerging reports, the crosstalk between γδ T17, microbiota and other cells reveals a complicated, dynamic network. Focusing on the regulatory factors driving IL-17 transcription in γδ T cells can help elucidate their role in cancer and could be the key to targeting γδ T17-mediated tumor growth and progression.

## Concluding Remarks

γδ T17 promote cancer progression through IL-17 via various mechanisms in murine models and some human cancers. Understanding the molecular and metabolic mechanisms orchestrating IL-17 production of γδ T cells provides us with a better understanding of how these cells are supposed to function in health and how disease alters these processes. Knowing the intricate mechanisms governing IL-17 production can help contribute to the development of new therapies targeting γδ T17-associated inflammation and tumor progression.

## Author Contributions

XC and SM drafted the manuscript. JY and FC discussed and revised the manuscript. All authors read and approved the final manuscript for submission.

### Conflict of Interest

The authors declare that the research was conducted in the absence of any commercial or financial relationships that could be construed as a potential conflict of interest.
